# Factors affecting elective cesarean section in women with multiple pregnancy in Caruban, Indonesia

**DOI:** 10.12688/f1000research.27292.2

**Published:** 2022-03-14

**Authors:** Muhammad Pradhiki Mahindra, Mahendra Tri Arif Sampurna, Muhammad Pradhika Mapindra, Apriska Mega Sutowo Putri, Aries Krisbiyantoro, Rozi Aditya Aryananda

**Affiliations:** 1 Maternal-Fetal Medicine Department, Elizabeth Garrett Anderson Institute for Women's Health, University College London, London, WC1E 6AU, UK; 2 Department of Pediatrics, Faculty of Medicine, Universitas Airlangga, Surbaya, East Java, 60115, Indonesia; 3 Neonatology Department, Elizabeth Garrett Anderson Institute for Women's Health, University College London, London, WC1E 6AU, UK; 4 Faculty of Medicine, Sebelas Maret University, Surakarta, East Java, 57126, Indonesia; 5 Caruban General Hospital, Madiun, East Java, 63153, Indonesia; 6 Department of Obstetrics and Gynecology, Airlangga University, Subaya, East Java, 60115, Indonesia

**Keywords:** cesarean section, complication, multiple pregnancy, vaginal birth

## Abstract

**Background**: Caesarean sections have become the most popular method for delivering twin babies because of the safety concerns associated with a natural birth. This study aims to identify the maternal characteristics and obstetric parameters that serve as risk factors influencing caesarean delivery in twin pregnancies by comparing women delivering via caesarean section and vaginal birth.

**Methods**: A retrospective chart review design was used to analyse 47 women with multiple pregnancies from the medical records at a primary referral hospital in East Java, Indonesia. Women delivering vaginally were then compared with women who underwent a caesarean section to identify any differences between the groups.

**Results**: In our study, more women delivered by caesarean section than by vaginal birth. Women were more likely to undergo a caesarean section if they had a previous history of undergoing a caesarean section. Similar to previous studies, we found that foetal malpresentation significantly increase the risk of caesarean delivery, while labour augmentation decrease the likelihood of caesarean section. There was also a significant difference in maternal age between groups.

**Conclusions**: The percentage of multiple pregnancies delivered via caesarean section is quite high. Other larger cohort study are warranted, since many factors were involved in the decision of caesarean section.

## Introduction

Multiple pregnancies are currently much more common than in the past as a result of older maternal age and pregnancies resulting from assisted reproductive technologies ^1^. Multiple pregnancy accounts for 1% to 4% of total births, with a prevalence ranging from 0.9% to 1.4% in England and Wales and 0.9% to 2.4% in Brazil ^2^. The lowest incidence rate of multiple pregnancy is in East Asia (fewer than eight twin births in 1000) ^3^. Multiple pregnancy is associated with higher maternal and perinatal risks than singleton pregnancy, including pre-eclampsia, hypertension, hyperemesis, postpartum haemorrhage, premature rupture of the membranes and anaemia ^2, 4^. The rate of perinatal mortality is 2–3 times higher in multiple pregnancy than in singleton newborns. Multiple pregnancy can increase preterm birth, foetal growth restriction, low birth weight and intrapartum anoxia ^5^.

A case complicated by multiple pregnancy has the same delivery options as a singleton pregnancy: caesarean or vaginal delivery. This choice depends on multiple factors and remains controversial in numerous studies. Many important factors are involved when determining the delivery method, including women’s parity, foetal weight, foetal presentation, inter-twin weight discordance and maternal clinical conditions ^6^. Some studies have explained that if both foetuses are in a cephalic presentation, the vaginal mode is preferred ^7^. Vaginal delivery is possible if the foetus weighs more than 1500 grams. Caesarean delivery is the best choice when the first foetus is non-cephalic. Another large retrospective population study found that compared with caesarean delivery, increased neonatal morbidity and mortality were observed for the second twin when vaginal delivery was performed. That study also indicated that elective caesarean delivery is safer than vaginal delivery and results in the best maternal and perinatal outcomes ^8^. However, although evidence for the preference of an elective caesarean section for multiple pregnancy is lacking, the rates of elective caesarean section for multiple pregnancy have increased throughout the world ^9^. The main objective of this study was to evaluate and provide reliable information on the contributing risk factors such as maternal characteristics, obstetric histories and recorded pregnancy complications that influenced the decision to undertake a caesarean section or vaginal birth in multiple pregnancy cases.

## Methods

### Study settings

This retrospective chart review investigated secondary data that were obtained from written medical records at Caruban General Hospital in Caruban, East Java, which has become one of the main primary referral hospitals that receives many high-risk pregnancy cases from the primary health centres and antenatal clinics in the region of Madiun.

### Study design and population

This analysis used a purposive sampling method from the records of pregnant women with multiple pregnancies in the hospital’s data registry from 1 December 2014, to 15 December 2019. We identified 95 medical records of pregnant women based on several criteria. We obtained the data of women who had experienced multiple pregnancy and undergone elective caesarean section or vaginal delivery in the Caruban and Madiun regions of East Java, Indonesia. The inclusion criteria were as follows: women older than 18 years without mental disorders and who were medically stable (oxygen level > 93%, heart rate 60–100 bpm, blood pressure less than 80/60 mmHg). The exclusion criteria were as follows: emergency caesarean section, medically unstable (oxygen level < 93%, heart rate below 60 or above 100 bpm, women with altered mental status), death of the newborn and death of the mother. However, there were no cases of neonatal or maternal death or injury. Medical records with incomplete and unclear information on maternal characteristics were not included. The final sample consisted of 47 women.

### Data collection and analysis

Using a data extraction form (see extended data ^10^), information on maternal characteristics including age, educational background and occupational status and information on obstetric histories such as previous history of caesarean section and parity status, were obtained. We decided to categorise educational background into graduates and unqualified degree. Occupational status was subcategorised into unemployed, civil servant, private employee and entrepreneur. Parity status was classified into nulliparous, primiparous and multiparous. Data on perinatal events including foetal malpresentation, labour augmentation, prenatal haemoglobin levels and gestational hypertension were also recorded (see underlying data for the full dataset ^11^).

The analyses were performed using the Statistical Package for the Social Sciences (SPSS) version 16 for Windows. Quantitative data are presented as odds ratios (ORs) with confidence intervals (CIs) and means with the standard deviation. Chi-square tests were used to compare the caesarean and vaginal birth groups, and normal probability plots were generated to evaluate the data distribution. We then used an ANOVA for the normally-distributed data and the Kruskal–Wallis test for the non-normally-distributed data. A P-value of <0.05 was considered significant.

### Ethics

This analysis was approved by the ethics committee of Caruban General Hospital (No. 800/ 4532/ 402.102.110/ 2020). The requirement to obtain informed consent was waived by the ethics committee of Caruban General Hospital before the study was conducted. The study was conducted according to the relevant guidelines. The patient data records were coded and anonymised. The information recorded is confidential and was used only for the study purpose.

## Results

**Figure 1.**
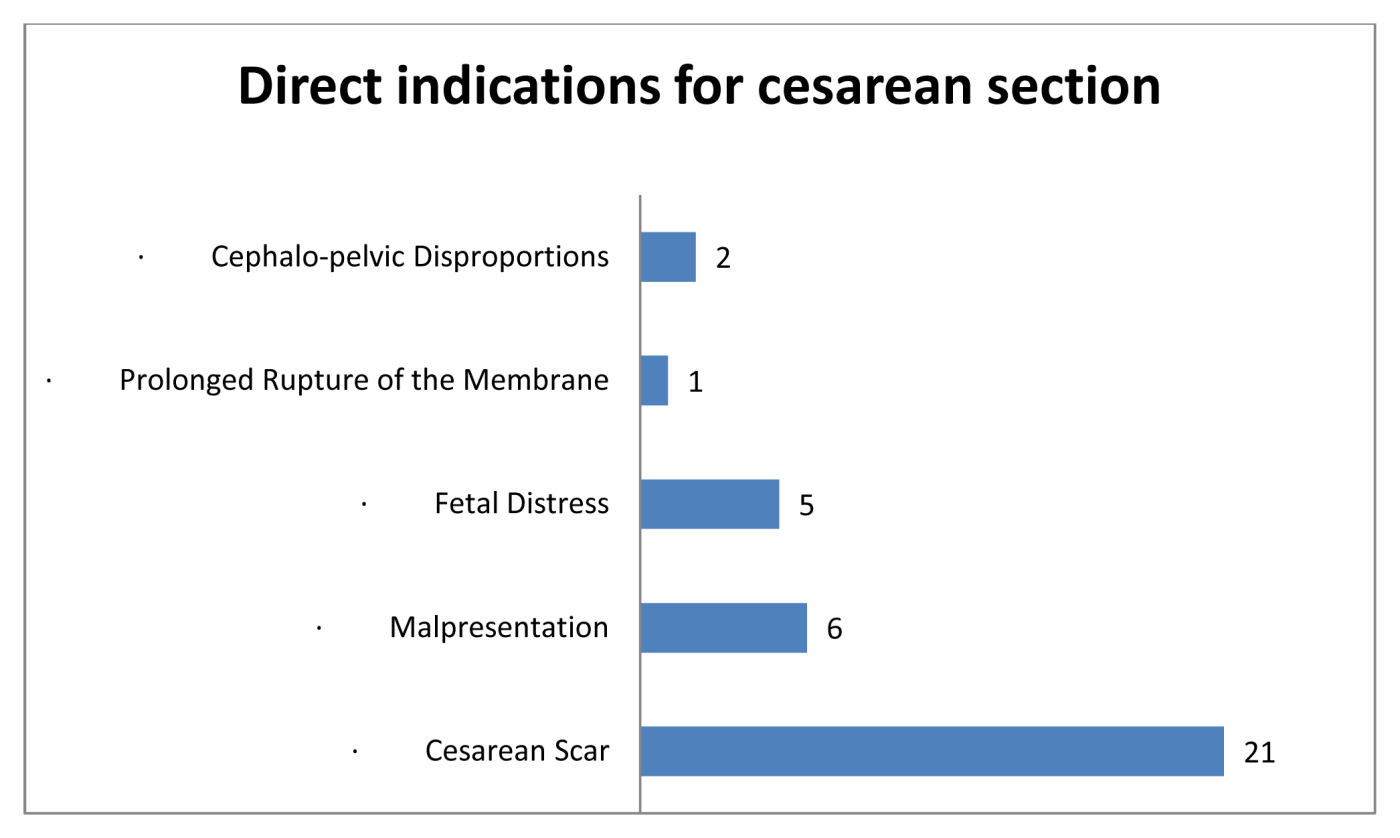
Cesarean section indications.

As shown in Table 1 ^11^, among the 47 mothers who met our inclusion criteria, more than half were between 21 and 35 years old, and most had a low educational status and worked as private employees. Additionally, 12 women underwent a vaginal delivery whereas the other 35 women birthed their babies abdominally. Thus, more women underwent a caesarean section than gave birth vaginally. Figure 1 indicates that the main indication for a caesarean section in this study was a caesarean scar, followed by malpresentation and foetal distress. Perinatal complications were principally dominated by malposition of the foetuses, which were not in the vertex position. During the labour process, 18 of the 47 women suffered prolonged labour. Six women came to the hospital with a history of premature membrane rupture without uterine contractions. Gestational hypertension was also reported in 12 women and 11 women had Hb levels below 10 g/dL in their last recorded Hb examinations in late pregnancy.

**Table 1.** Demographic and obstetrical data.

Variables	Method of delivery		OR (95%CI)	P-Value
	Section Cesarean	Vaginal Birth		
	35	12		
**Parity Status, n (%)**				
Nulliparous	6 (17.1%)	5 (41.7%)	0.29 (0.06,1.22)	0.12
Primiparous	21 (60.0%)	6 (50.0%)	1.50 (0.40,5.60)	0.73
Multiparous	8 (22.9%)	1 (8.3%)	3.25 (0.36, 29.23)	0.41
**Age, median (min, max)**	30 (20, 39)	20 (19, 37)	1.26 (1.09,1.45)	**0.00**
**Educational Status, n (%)**				
Unqualified degree	9 (25.7 %)	5 (41.7%)	0.48 (0.12,1.91)	0.46
Graduates	26 (74.3%)	7 (58.3%)	1	
**Occupation, n (%)**				
Unemployed	11 (31.4%)	3 (25.0%)	1.37 (0.31,6.09)	1.00
Civil Servant	9 (25.7%)	4 (33.3%)	0.69 (0.16,2.86)	0.71
Private employee	10 (28.6%)	4 (33.3%)	0.80 (0.19, 3.26)	0.73
Entrepreneur	5 (14.3%)	1 (8.3 %)	1.83 (0.19 17.48)	1.00
**Previous History of Section, n (%)**				
Yes	21 (60.0%)	1 (8.3%)	16.5 (1.91,142.49)	**0.02**
No	14 (40.0%)	11 (91.7%)	1	
**Foetal presentation, n (%)**				
Malpresentation	15 (42.9 %)	1 (8.3%)	8.25 (0.95, 71.09)	**0.03**
Normal	20 (57.1%)	11 (91.7 %)	1	
**Augmentation of Labor, n (%)**				
Yes	10 (28.6%)	8 (66.7%)	0.20 (0.49, 0.81)	**0.03**
No	25 (71.4%)	4 (33.3%)	1	
**Premature Rupture of the Membrane, n (%)**				
Yes	6 (100 %)	0 (0.0 %)	0.82 (0.71, 0.96)	0.31
No	29 (70.7%)	12 (100 %)	1	
**Gestational Hypertensive Disorders, n (%)**				
Yes	11 (31.4%)	1 (8.3%)	5.04 (0.57, 44.06)	0.14
No	24 (68.6%)	11 (91.7%)	1	
**Prenatal Hemoglobin Levels,** **mg/dl (Mean ± SD)**	11.18 ± 1.69	10.80 ± 1.11		0.48
Gestational Anemia **, n (%)**	9 (25.7%)	2 (16.7%)	1.73 (0.31, 9.44)	0.70
Non-anemia **, n (%)**	26 (74.3%)	10 (83.3%)	1	
**Delivery at Gestational Age,** **weeks, Median (min, max)**	37 (34, 37)	37 (31, 38)		0.55
< 37 weeks **, n (%)**	14 (40.0%)	5 (41.7%)	1	1.00
37–42 weeks **, n (%)**	21 (60.0%)	7 (58.3 %)	0.93 (0.24, 3.53)	

Based on the analysis, the average age at delivery differed significantly between women who underwent caesarean and vaginal delivery (p = 0.00). Other maternal characteristics including primiparous as the most common parity status (p = 0.73), educational status (p = 0.46) and being unemployed (p = 1.00) did not differ significantly between the two groups of women stratified by delivery method.

Additionally, we evaluated the obstetric history of the included mothers. In total, 21 women had a history of previous caesarean section, which was found to significantly increase the likelihood of undergoing another caesarean section (OR = 16.5, 95% CI = 1.91 to 142.29, p = 0.02). Women who suffered prolonged labour during their delivery were significantly less likely to undergo a caesarean delivery (OR = 0.20, 95% CI = 0.49 to 0.81, p = 0.03). Women who experience malpresentation of their foetuses during pregnancy were more likely to undergo a caesarean delivery (OR = 8.25, 95% CI = 0.95, 71.09, p = 0.03). However, in this study, premature rupture of the membrane, gestational hypertension and gestational anaemia did not significantly contribute to an increased likelihood of a caesarean section. Pregnancy term did not differ significantly between the women undergoing caesarean section and vaginal birth. Neonatal outcomes including 1-minute APGAR (appearance, pulse, grimace, activity, and respiration) scores and babies’ birth weights did not differ significantly for both the first and second babies between both groups of delivery methods ( Table 2 ^11^). When comparing caesarean section and vaginal delivery, both first babies (p = 0.82) and second babies (p = 0.38) had APGAR scores ≥ 7 ( Table 2 ^11^). The first babies delivered after caesarean section vs vaginal birth did not differ significantly (2114 grams ± 469.37 vs 1938.33 grams ± 562.36, p = 0.29) ( Table 2 ^11^). Similarly, the second babies following caesarean section vs vaginal birth also did not differ significantly (2038 ± 478.75 vs 1878.33 ± 417.45, p = 0.31) ( Table 2 ^11^).

**Table 2.** Comparison of neonatal parameters between women undergoing cesarean section and vaginal birth.

Parameters	Method of delivery		p-value
	Cesarean Section 35	Vaginal Birth 12	
**Neonatal characteristics**			
Gender			
First Baby, n (%)			
Male	14 (40)	8 (66.7)	0.18
Female	21 (60)	4 (33.3)	
Second Baby			
Male	15 (42.9)	7 (58.3)	0.55
Female	20 (57.1)	5 (41.7)	
1-minute APGAR score, median (min, max)			
First Baby	8 (6, 8)	7 (7, 8)	0.82
Second Baby	7 (5, 8)	7 (7,8)	0.38
Birth Weight, (Mean ± SD) gram			
First Baby	2114 ± 469.37	1938.33 ± 562.36	0.29
Second Baby	2038 ± 478.75	1878.33 ± 417.45	0.31

APGAR: appearance, pulse, grimace, activity, and respiration

## Discussion

Based on the study results, a planned caesarean section appears preferable to a vaginal birth for women with multiple pregnancies when the mothers have a history of previous caesarean section and in cases of malpresentation of the foetus. The neonatal birth weight and 1-minute APGAR scores of the twin babies born by both methods of delivery in this study were similar. Adverse perinatal outcomes including perinatal mortality and transient tachypnea of the newborn, were not significantly different between groups. However, the second twin had a higher risk of suffer perinatal outcomes than the first following vaginal delivery ^12^. Combined vaginal/caesarean delivery is another option. However, it may be complicated by a longer interval of prolonged rupture of membranes, leading to endometritis and sepsis in the second twin. ^13^. A large nationwide study of multiple pregnancy cases identified a greater risk of uterine rupture in women who delivered their babies via natural birth ^14^. However, previous studies reported that there was no difference in the perinatal outcomes between caesarean section and vaginal birth in multiple pregnancy ^15, 16^. Conversely, maternal aspects are an important consideration when choosing the delivery mode in women with a twin pregnancy. However, some studies have reported that the use of forceps or vacuum extractions in vaginal birth could contribute to birth lacerations that may lead to gynaecologic morbidities ^17– 20^.

In this comparison study between planned caesarean section and vaginal delivery, we found that there was a significant difference in the maternal age between the two groups. This finding was supported by a previous study that showed a significantly increased trend of caesarean delivery with increased maternal age ^21^. However, a study from Korea found there was no significant difference caesarean section rates in women with multiple pregnancies according to maternal age ^22^. The other difference we identified was that women undergoing caesarean section were more likely to have undergone a previous caesarean section. A similar study comparing women having caesarean sections and vaginal births reported that caesarean section was significantly more likely to occur in women with previous caesarean histories ^23^. Another report revealed that caesarean sections must not be considered mandatory for multiple pregnancies ^24^. However, a study from Myles (2009) demonstrated that caesarean sections had a higher success rate and a lower probability of developing uterine rupture than vaginal delivery ^25^.

This study found that women who underwent labour augmentation by using misoprostol or oxytocin did not have a higher probability of caesarean section. Supporting our finding, a study by Arulkumaran showed that approximately 78% of women with a caesarean scar who were administered labour augmentation had safe vaginal births whereas the other women who had undergone second caesarean sections were suffering from cephalo-pelvic disproportion ^26^. Furthermore, a prospective cohort study on 153 women with caesarean scars in Saudi Arabia suggested that labour induction to promote vaginal delivery did not contribute to the increased likelihood of a second elective caesarean section or adverse effects on neonatal outcomes ^27^. By contrast, a study in Sweden reported the increased likelihood of caesarean delivery after performing labour induction using oxytocin and cervical ripening in women with multiple pregnancy ^28^. Another study also explained that labour induction or augmentation was safe for women to promote successful vaginal delivery in multiple pregnancy cases ^29^.

In our study, we found that non-cephalic presentations did not significantly increase the likelihood of undergoing a caesarean section. Our results supported previous studies that reported relatively similar outcomes for women with non-cephalic presentations who delivered their babies abdominally and vaginally ^30, 31^. Furthermore, a caesarean section in multiple pregnancies with non-cephalic presentations is likely to occur following external cephalic attempts ^11^. A report from France suggested that the type of presentation must not be considered the main consideration for caesarean section in multiple pregnancies ^10^.

Our findings demonstrate that women with multiple pregnancies had a higher tendency to deliver their babies abdominally via caesarean section. Maternal age and previous history of a caesarean section should be considered when determining whether to perform a caesarean delivery in women with multiple pregnancies. However, the supporting findings are still limited. This study also noted that labour augmentation decrease the likelihood of a caesarean section compared with vaginal delivery. However, this finding was rather weak due to the small size of the study population, and thus choosing between a caesarean section and vaginal delivery must be made after considering the patient’s preference and the risks and benefits.

Several others limitation exist in our study, due to small sample size, the data may not reach all others contributing factors. Therefore it is difficult to get a reliable insight from this retrospective study about the contributing risk factors for caesarian section in multiple pregnancy. Obstetricians have a variety of reasons for performing a caesarean section, which are not always linked to real risk factors for the woman or the fetus. There are may be some subjective reason in choosing the caesarean section. However, our findings can serve as a mirror for obstetricians to consider the clear objective justifications for performing a caesarean procedure in a twin pregnancy.

## Data availability

### Underlying data

Figshare: Underlying Data - Factors Affecting Elective Cesarean Section in Women with Multiple Pregnancy at a Primary Referral Hospital in Indonesia. https://doi.org/10.6084/m9.figshare.13166735.v1 ^11^

This project contains the following the underlying data:

- Cesarean Section VS Vaginal Birth (Maternal Factors).sav
- Cesarean Section VS Vaginal Birth (Neonatal Factors).sav

### Extended data

Figshare: Data Extraction Form - Factors Affecting Elective Cesarean Section in Women with Multiple Pregnancy at a Primary Referral Hospital in Indonesia. https://doi.org/10.6084/m9.figshare.13238534.v1 ^10^

 Data are available under the terms of the Creative Commons Attribution 4.0 International license (CC-BY 4.0).
