# Supplementary material for: Factors affecting elective cesarean section in women with multiple pregnancy in Caruban, Indonesia
Source: F1000Res. 2023 Aug 10;9:1481. Originally published 2020 Dec 18. [Version 3] doi: 10.12688/f1000research.27292.3 (PMC10724646; doi:10.12688/f1000research.27292.3)
Supplement: Peer review history

# Reviewer report: Reviewer response for version 3

**DOI:** 10.5256/f1000research.153085.r195442

**Published:** 2023-12-15

**Authors:** Kian Djien Liem

**Affiliations:** 1 Department of Neonatology, Amalia Children’s Hospital, Nijmegen, The Netherlands

The authors have satisfactorily improved the manuscript.

Is the work clearly and accurately presented and does it cite the current literature?

Yes

If applicable, is the statistical analysis and its interpretation appropriate?

Partly

Are all the source data underlying the results available to ensure full reproducibility?

Yes

Is the study design appropriate and is the work technically sound?

Yes

Are the conclusions drawn adequately supported by the results?

Yes

Are sufficient details of methods and analysis provided to allow replication by others?

Yes

Reviewer Expertise:

Neonatology

I confirm that I have read this submission and believe that I have an appropriate level of expertise to confirm that it is of an acceptable scientific standard.

# Reviewer report: Reviewer response for version 3

**DOI:** 10.5256/f1000research.153085.r195443

**Published:** 2023-08-24

**Authors:** Gatot Nyarumenteng Adhipurnawan Winarno, Ayu Insafi Mulyantari

**Affiliations:** 1 Department Obstetric and Gynecology, Universitas Padjadjaran, Bandung, West Java, Indonesia; 2 Department of Obstetrics and Gynecology, Dr. Hasan Sadikin Central General Hospital, Faculty of Medicine, Universitas Padjadjaran, Bandung, Indonesia

The authors already addressing the items brought up during the prior round of review.

Is the work clearly and accurately presented and does it cite the current literature?

Yes

If applicable, is the statistical analysis and its interpretation appropriate?

Yes

Are all the source data underlying the results available to ensure full reproducibility?

Yes

Is the study design appropriate and is the work technically sound?

Yes

Are the conclusions drawn adequately supported by the results?

Yes

Are sufficient details of methods and analysis provided to allow replication by others?

Partly

Reviewer Expertise:

Obstetrics and Gynecology

We confirm that we have read this submission and believe that we have an appropriate level of expertise to confirm that it is of an acceptable scientific standard.

# Reviewer report: Reviewer response for version 2

**DOI:** 10.5256/f1000research.119919.r172973

**Published:** 2023-07-12

**Authors:** Gatot Nyarumenteng Adhipurnawan Winarno, Ayu Insafi Mulyantari

**Affiliations:** 1 Department of Obstetrics and Gynecology, Dr. Hasan Sadikin Central General Hospital, Faculty of Medicine, Universitas Padjadjaran, Bandung, Indonesia; 2 Department Obstetric and Gynecology, Universitas Padjadjaran, Bandung, West Java, Indonesia

**Abstract:**

 I encourage the authors to add the exact numbers in the result section, such as p-value, OR etc

**Method**

 In the method section, the author states that BP less than 80/60 was the inclusion criteria. How can this be? The patients should be in a shock state, which is not eligible as the study object. 

**Result**

 The author state that the *"more than half were between 21 and 35 years old, and most had a low educational status and worked as private employees*" Please revisit your table presentation. This data was not matched with the data you're presenting in Table 1. Both for age and occupation (the table presented that 11 study object was unemployed). 

**Table 1**

 Correct me if I am mistaken, the author presents the parity as nulliparous, primiparous, and multiparous. There are 21 study objects that are classified as primiparous. Meanwhile, there are 21 study objects which had the previous history of section. This might lead the ambiguity or mislead the reader. Thus, the classification for parity might be changing into primiparous (n=6) and multiparaous (n=29)

Is the work clearly and accurately presented and does it cite the current literature?

Yes

If applicable, is the statistical analysis and its interpretation appropriate?

Yes

Are all the source data underlying the results available to ensure full reproducibility?

Yes

Is the study design appropriate and is the work technically sound?

Yes

Are the conclusions drawn adequately supported by the results?

Yes

Are sufficient details of methods and analysis provided to allow replication by others?

Partly

Reviewer Expertise:

Obstetrics and Gynecology

We confirm that we have read this submission and believe that we have an appropriate level of expertise to confirm that it is of an acceptable scientific standard, however we have significant reservations, as outlined above.

# Response

**Published:** 2023-07-15

**Authors:** Mahendra Tri Arif Sampurna

**Affiliations:** Faculty of Medicine Universitas Airlangga, Indonesia

We are very thankful for your availability and willingness to help us to become our reviewer on our manuscript. We have revised our manuscript based on point-on-point revision as suggested.

 1. Abstract: I encourage the authors to add the exact numbers in the result section, such as p-value, OR etc

**Response:** Thank you for your suggestion, we are very much agreed with your suggestion and have put the exact number on the abstract.

 2. Method: In the method section, the author states that BP less than 80/60 was the inclusion criteria. How can this be? The patients should be in a shock state, which is not eligible as the study object. 

**Response:** Thank you for your correction, we realize that there is an error information and have deleted it from the manuscript.

 3. Result: The author state that the "more than half were between 21 and 35 years old, and most had a low educational status and worked as private employees" Please revisit your table presentation. This data was not matched with the data you're presenting in Table 1. Both for age and occupation (the table presented that 11 study object was unemployed).

**Response: **Thank you for your correction, we realize that there is an error information and have revised it.

 4. Table 1 : Correct me if I am mistaken, the author presents the parity as nulliparous, primiparous, and multiparous. There are 21 study objects that are classified as primiparous. Meanwhile, there are 21 study objects which had the previous history of section. This might lead the ambiguity or mislead the reader. Thus, the classification for parity might be changing into primiparous (n=6) and multiparaous (n=29)

**Response: **Thank you for your suggestion, we agreed that statement is ambiguous, but because of our aim to search for factors, we classified the parity by the condition before delivery (nulliparous : 0; primiparous : 1; multiparous > 2), we will add some explanation on the definition in the methods section to avoid ambiguation.

# Reviewer report: Reviewer response for version 2

**DOI:** 10.5256/f1000research.119919.r127395

**Published:** 2022-07-26

**Authors:** Kian Djien Liem

**Affiliations:** 1 Department of Neonatology, Amalia Children’s Hospital, Nijmegen, The Netherlands

The authors have satisfactorily corrected the conflicting data on fetal malpresentation.

 They have also mentioned the limitations of their study.

 However, there are still a few points that need some clarification.

- In the abstract in the last sentence: " *There was also a significant difference in maternal age between groups.". *This is very vague. It is not clear which significant difference is found. This is also the case in the results. It is only mentioned: *"the average age at delivery differed significantly between women who underwent caesarean and vaginal delivery (p = 0.00)". *But for clarity it is desirable to indicate in what sense there is a difference.
- In the results, the last paragraph: " *The first babies delivered after caesarean section vs vaginal birth did not differ significantly (2114 grams ± 469.37 vs 1938.33 grams ± 562.36, p = 0.29) (Table 2 ^11^). Similarly, the second babies following caesarean section vs vaginal birth also did not differ significantly (2038 ± 478.75 vs 1878.33 ± 417.45, p = 0.31) (Table 2 ^11^)." *It is not stated for which variable the difference is analyzed. Based on the results, it seems to be the birth weight. Therefore, for the clarity for the readers it is desirable to mention that it concerns birth weight.
- In the discussion, in the first paragraph the authors summarize their findings, but after the sentence: *"Adverse perinatal outcomes including perinatal mortality and transient tachypnea of the newborn, were not significantly different between groups",* they mentioned: *"However, the second twin had a higher risk of suffer perinatal outcomes than the first following vaginal delivery" *and referring to reference no. 12. This might cause some confusion to the reader. In the results the authors didn't find a significant difference between the first and the second baby according to the neonatal outcome in both groups of delivery methods. Therefore it is better to mention: However, this is in contradictory to the previous study, where the second twin had a higher risk of adverse perinatal outcomes than the first following vaginal delivery and referring this to the reference no. 12.

Is the work clearly and accurately presented and does it cite the current literature?

Yes

If applicable, is the statistical analysis and its interpretation appropriate?

Partly

Are all the source data underlying the results available to ensure full reproducibility?

Yes

Is the study design appropriate and is the work technically sound?

Yes

Are the conclusions drawn adequately supported by the results?

Yes

Are sufficient details of methods and analysis provided to allow replication by others?

Yes

Reviewer Expertise:

Neonatology

I confirm that I have read this submission and believe that I have an appropriate level of expertise to confirm that it is of an acceptable scientific standard, however I have significant reservations, as outlined above.

# Response

**Published:** 2023-07-15

**Authors:** Mahendra Tri Arif Sampurna

**Affiliations:** Faculty of Medicine Universitas Airlangga, Indonesia

We are very thankful for your availability and willingness to help us to become our reviewer on our manuscript. We have revised our manuscript based on point-on-point revision as suggested.

 1. In the abstract in the last sentence: "There was also a significant difference in maternal age between groups.". This is very vague. It is not clear which significant difference is found. This is also the case in the results. It is only mentioned: "the average age at delivery differed significantly between women who underwent caesarean and vaginal delivery (p = 0.00)". But for clarity it is desirable to indicate in what sense there is a difference.

**Response: **We are very thankful for your suggestion, we have revised this issue in the manuscript.

 2. In the results, the last paragraph: "The first babies delivered after caesarean section vs vaginal birth did not differ significantly (2114 grams ± 469.37 vs 1938.33 grams ± 562.36, p = 0.29) (Table 211). Similarly, the second babies following caesarean section vs vaginal birth also did not differ significantly (2038 ± 478.75 vs 1878.33 ± 417.45, p = 0.31) (Table 211)." It is not stated for which variable the difference is analyzed. Based on the results, it seems to be the birth weight. Therefore, for the clarity for the readers it is desirable to mention that it concerns birth weight.

**Response:** We are very thankful for your comments, we acknowledge that this is not clear enough, therefore we have mentioned the concern about birth weight.

 3. In the discussion, in the first paragraph the authors summarize their findings, but after the sentence: "Adverse perinatal outcomes including perinatal mortality and transient tachypnea of the newborn, were not significantly different between groups", they mentioned: "However, the second twin had a higher risk of suffer perinatal outcomes than the first following vaginal delivery" and referring to reference no. 12. This might cause some confusion to the reader. In the results the authors didn't find a significant difference between the first and the second baby according to the neonatal outcome in both groups of delivery methods. Therefore it is better to mention: However, this is in contradictory to the previous study, where the second twin had a higher risk of adverse perinatal outcomes than the first following vaginal delivery and referring this to the reference no. 12.

**Response:** We are very thankful for your suggestion, we very much agreed and have revised it.

# Reviewer report: Reviewer response for version 1

**DOI:** 10.5256/f1000research.30156.r76524

**Published:** 2021-02-02

**Authors:** Kian Djien Liem

**Affiliations:** 1 Department of Neonatology, Amalia Children’s Hospital, Nijmegen, The Netherlands

**General comment:**

 This is a retrospective study which has the intention to identify the contributing risk factors for the decision of performing caesarean section in twin pregnancies. It is very difficult to get a reliable insight from this retrospective study about the contributing risk factors for caesarian section in multiple pregnancy. There are many reasons of the obstetricians for choosing caesarean section, which are not always necessary correlated with some real risk factors for the mother or the foetus. Possibly that some of these reasons are subjectively determined. Therefore it is not surprising to find that fetal malpresentation is not more common in the caesarean section group as compared to the group of vaginal delivery. Nevertheless, the result of this retrospective study can be used as a mirror for the obstetricians in order to think about the clear objective indications for performing of caesarean section in twin pregnancy. The authors should make an obstetrical recommendation for a reliable protocol of caesarean section indication in twin pregnancy.

**Specific comments:**

Abstract:

- No comment.

Introduction:

- Line 6 from the second paragraph: what is meant by gestational weight? Is this maternal weight or estimated fetal weight? The authors must clarify this.

Methods:

- No comment.

Results:

- Table 1 - This part of the table is incorrect: Under vaginal birth is 1 of 12 never 66.7% and 11 of 12 is never 42.9%. The total percentage is more than 100%. Probably the OR and p-value has to be recalculated again.

- Also in the text under the 3 ^rd^ paragraph, the OR an p-value has to be checked also. I am wondering why fetal malpresentation didn’t result in more cesarean section. Especially when some fetal malpresentation will result in difficult either impossible vaginal delivery.

Discussion:

- In the first line the authors make the following statement: “Based on the study results, a planned caesarean section appears preferable to vaginal birth for women with multiple pregnancies when the mothers have a history of previous caesarean section and in cases of malpresentation of the fetus”. But this seems in contradiction with their results that women who did not experience malpresentation of their fetuses during pregnancy were more likely to undergo a caesarean delivery. The authors should clarify this contradiction.
- In the first paragraph the authors write: “However the second twin had a higher risk of suffer perinatal outcomes than the first following vaginal delivery”. But this statement is not supported by the data of their results.
- In this chapter a discussion about the limitation of this study is missed. The authors should make a reflection about the limitations of their study. See also general comment.

Is the work clearly and accurately presented and does it cite the current literature?

Yes

If applicable, is the statistical analysis and its interpretation appropriate?

Partly

Are all the source data underlying the results available to ensure full reproducibility?

Yes

Is the study design appropriate and is the work technically sound?

Yes

Are the conclusions drawn adequately supported by the results?

Yes

Are sufficient details of methods and analysis provided to allow replication by others?

Yes

Reviewer Expertise:

Neonatology

I confirm that I have read this submission and believe that I have an appropriate level of expertise to confirm that it is of an acceptable scientific standard, however I have significant reservations, as outlined above.
